# A quick algorithmic review on management of viral infectious diseases in pediatric solid organ transplant recipients

**DOI:** 10.3389/fped.2023.1252495

**Published:** 2023-09-04

**Authors:** Marjan Moghadamnia, Hamid Eshaghi, Hosein Alimadadi, Simin Dashti-Khavidaki

**Affiliations:** ^1^ Department of Pharmacotherapy, Faculty of Pharmacy, Tehran University of Medical Sciences, Tehran, Iran; ^2^ Department of Infectious Diseases, Pediatrics’ Center of Excellence, Children’s Medical Center, Tehran University of Medical Sciences, Tehran, Iran; ^3^ Department of Gastroenterology, Children’s Medical Center, Tehran University of Medical Science, Tehran, Iran; ^4^ Department of Pharmacotherapy, Liver Transplantation Research Center, Tehran University of Medical Sciences, Tehran, Iran

**Keywords:** pediatrics, solid organ, transplantation, viral infections, antiviral

## Abstract

Pediatric solid organ transplant is a life-saving procedure for children with end-stage organ failure. Viral infections are a common complication following pediatric solid organ transplantation (SOT), which can lead to increased morbidity and mortality. Pediatric solid organ transplant recipients are at an increased risk of viral infections due to their immunosuppressed state. The most commonly encountered viruses include cytomegalovirus (CMV), Epstein-Barr virus (EBV), herpes simplex virus (HSV), varicella-zoster virus (VZV), adenoviruses, and BK polyomavirus. Prevention strategies include vaccination prior to transplantation, post-transplant prophylaxis with antiviral agents, and preemptive therapy. Treatment options vary depending on the virus and may include antiviral therapy and sometimes immunosuppression modification. This review provides a Quick Algorithmic overview of prevention and treatment strategies for viral infectious diseases in pediatric solid organ transplant recipient.

## 1. Introduction

Organ transplantation is a life-saving procedure for children suffering from end-stage organ diseases (1). Immunosuppressive therapy is necessary to prevent the rejection of transplanted organs in children; However, this therapeutic approach renders them highly susceptible to infections (2, 3). The probability of exposure to pathogens prior to transplantation and subsequent frequency of post-transplant infections can be influenced by the age of the patient at the time of the transplantation, the set of vaccinations received, and the intensity of immunosuppression (4, 5). The prevalence of seronegative recipients in the pediatric population is higher than in adults. Therefore, children are more sensitive to acquiring primary viral infections like CMV and EBV post-transplantation (6, 7).

Despite improvements in managing post-transplant infections during past 20 years, viral infections remain a significant factor affecting graft function and overall transplantation outcomes. Among this vulnerable population, delayed detection and management of viral infections can lead to significant levels of morbidity and mortality (8, 9). The most commonly observed viral infections following solid organ transplantation include CMV, EBV, HSV, VZV, adenoviruses, BK polyomavirus, and respiratory viruses like Influenza (10, 11).

Viral infections can have direct effects such as meningitis, pneumonia, encephalitis, and enteritis that adversely affect the patient's health condition. Moreover, viral infections can indirectly alter the immune system by inducing the release of chemokines and growth factors (12). Therefore, it is vital to closely monitor patients for identifying risk factors and providing preemptive therapy or prophylactic treatment in line with the center's policy (8).

This manuscript provides a quick, algorithmic overview on prevention and treatment of commonly encountered viral infections in post-solid organ transplant pediatric patients. With the implementation of these algorithms, we aim to reduce morbidity and mortality associated with these viral infections and to improve the long-term outcomes for pediatric organ recipients.

## 2. Cytomegalovirus

### 2.1. Prevention

**Figure 1:**
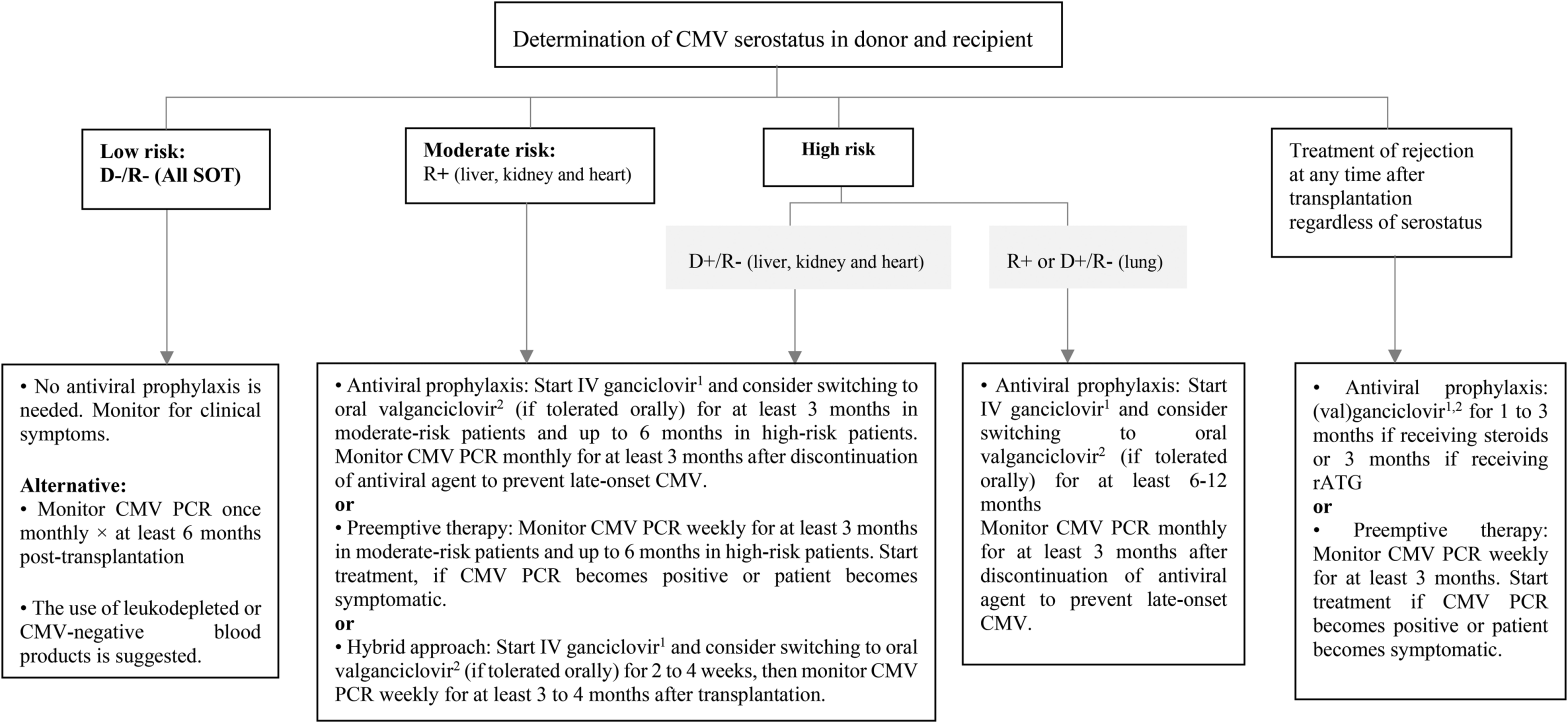
Prevention of CMV infection after pediatric SOT. CMV, cytomegalovirus; D, donor; IV, intravenous; PCR, polymerase chain reaction; R, recipient; rATG, rabbit antithymocyte globulin; SOT, solid organ transplantation. ^1^5 mg/kg/dose once daily in normal kidney function. ^2^Dose (mg): 7 × BSA × CrCl once daily.

During the pre-prophylaxis period, the incidence of CMV infection and disease among SOT recipients was high, while a great percentage of them was invasive (13). Recently, the use of measures such as antiviral prophylaxis, preemptive therapy, or hybrid strategies has led to a reduction in the incidence of CMV disease in pediatric SOT recipients (14–16). Antiviral prophylaxis involves using maintenance doses of (Val)ganciclovir starting within 10 days after transplantation for a duration of 3–6 months. Preemptive therapy is based on routine CMV polymerase chain reaction (PCR) monitoring and the administration of antiviral medication once viremia goes above a certain threshold to prevent CMV disease (16, 17). The hybrid approach involves a short course of antiviral prophylaxis (2–4 weeks), followed by viral load surveillance (15, 18). There have been insufficient pediatric studies to compare the efficacy of these three methods. Retrospective data show equal support for these three preventative approaches, hence all three are suggested (17, 19). However, a key factor for selecting the preventative method is the serostatus of the donor and recipient at the time of transplantation. There is a challenge in determining donor and recipient serostatus in infants younger than 12 months because the passive transfer of maternal antibodies may result in false positive results. According to the Third International Consensus Guidelines, risk assessment in this age group should be based on the highest level of risk for the purposes of CMV prevention (Table 1) (19). In low-risk SOT patients (D−/R−), CMV prophylaxis is not recommended as long as patients are not treated with leukocyte-depleting drugs and receive CMV-negative blood products. These patients should be monitored for clinical symptoms. Preemptive therapy can also be considered as an alternative in this group of patients because they are more prone to acquiring *de novo* CMV infection from exposures in the community (19). In moderate to high-risk liver, kidney and heart transplant recipients (R+ or D+/R−), all three methods of prevention can be adopted, but some centers prefer prophylaxis with antiviral agents in high-risk patients (D+/R−) (20, 21). In high-risk lung transplant recipients (R+ or D+/R−) antiviral prophylaxis is recommended for longer duration (at least 6–12 months) (19). Prevention with either antivirals or preemptive therapy is also recommended during the use of lymphocyte-depleting agents or intravenous steroids for treatment of acute allograft rejection (Figure 1).

**Table 1:** Serostatus assignment for donors and recipients in infants younger than 12 months.

Donor	Recipient	Classification of risk at the highest level
+	+ or −	D+/R−
−	+	D−/R+
−	−	D−/R−

### 2.2. Treatment

**Figure 2:**
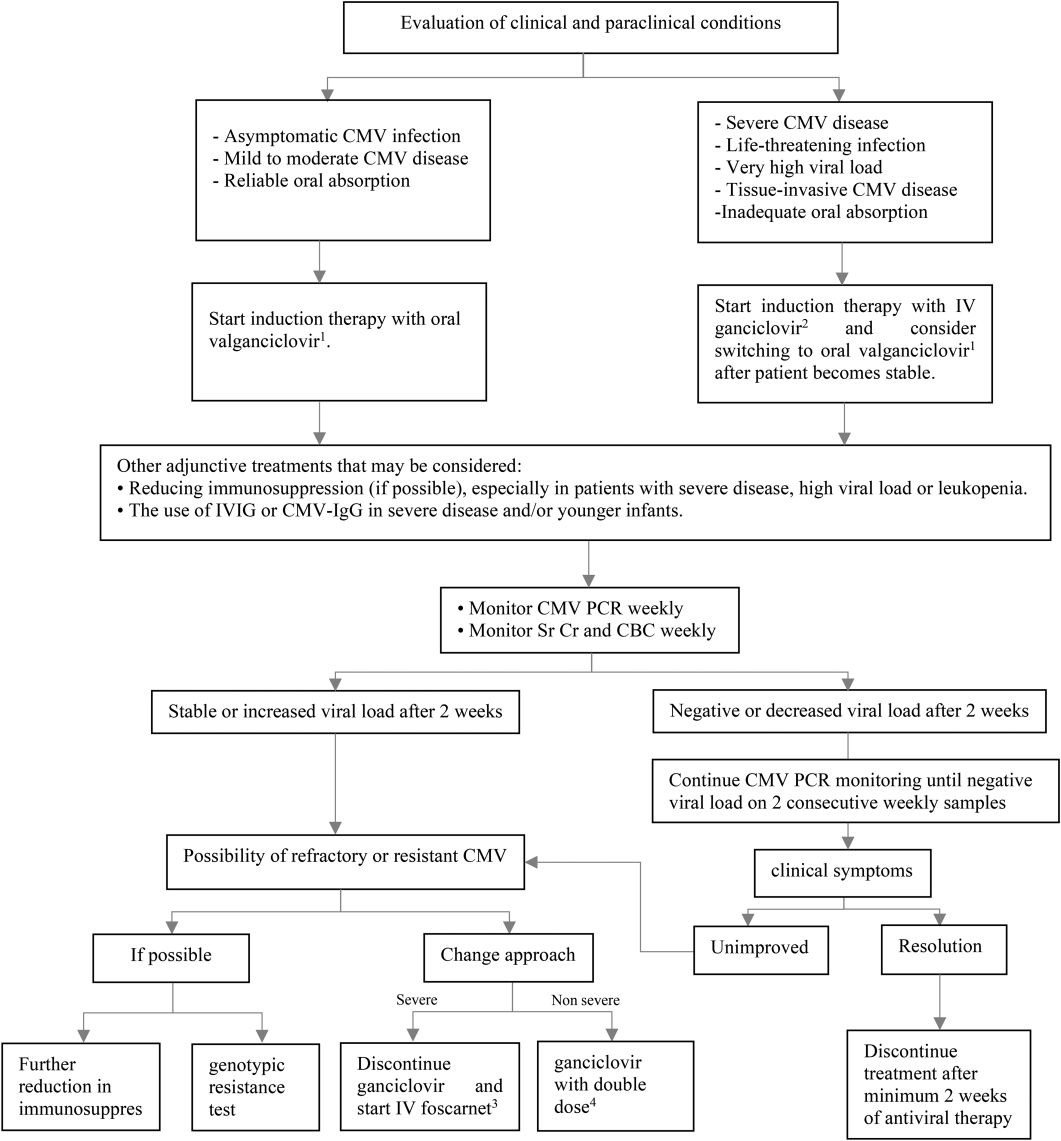
Treatment of CMV infection or disease after pediatric SOT. CBC, complete blood count; CMV, cytomegalovirus; IgG, immunoglobulin G; IV, intravenous; IVIG, intravenous immunoglobulin; PCR, polymerase chain reaction; SOT, solid organ transplantation; Sr Cr, serum creatinine. ^1^Dose (mg): 7 × BSA × CrCl twice daily. ^2^5 mg/kg/dose twice daily in normal kidney function. ^3^60 mg/kg/dose every 8 h OR 90 mg/kg/dose twice daily in normal kidney function. ^4^10 mg/kg/dose twice daily in normal kidney function.

In patients with reliable gastrointestinal absorption, oral valganciclovir is preferred for mild to moderate CMV disease or asymptomatic CMV infection (22). However, in the situations of severe or life-threatening CMV disease, very high viral load and tissue-invasive CMV disease, intravenous ganciclovir is recommended to rapidly achieve optimal drug levels. In clinically stable patients with declining and well-controlled viremia and resolved or resolving clinical symptoms, intravenous therapy can be switched to oral valganciclovir (Figure 2) (17). The dose of intravenous ganciclovir as initial therapy is 5 mg/kg every 12 h, which should be adjusted according to the level of kidney function. A dosing algorithm based on body surface area (BSA) and kidney function is recommended for valganciclovir (23). Patient's monitoring during antiviral therapy includes complete blood counts to check for hematologic side effects, kidney function assessments to determine antiviral dose adjustment, and weekly quantitative CMV PCR to assess medication response (22). Antiviral therapy should be continued as long as all three following conditions are met (17):

• Clinical symptoms subside.
• Virologic clearance to below a negative value threshold based on weekly CMV PCR.
• Antiviral therapy has been given for a minimum of two weeks.

In general, CMV-immune globulin (IgG) or intravenous immunoglobulin (IVIG) is not advised but may be used in conjunction with intravenous ganciclovir to treat CMV disease in young infants and more severe forms of the disease (17).

Although secondary prophylaxis is not currently supported by good-quality data, some centers offer it after completion of treatment for 1–3 months to lower the chance of recurrence (24). Secondary prophylaxis may be helpful for children with recurrent CMV DNAemia or disease (≥2 episodes). The length of secondary prophylaxis is influenced by patient's age, immunosuppressive regimen, being infected with other opportunistic infections, and other risk factors (19).

### 2.3. Antiviral-resistant CMV

According to pediatric cohorts studies the incidence of ganciclovir-resistance CMV is low. Empiric therapy includes doubling dose of ganciclovir or switching to full dose of foscarnet based on the severity of the disease (19). The UL97 kinase and UL54 DNA polymerase are two gene mutations that are associated with CMV drug resistance. Based on results of genotypic test, other antiviral agents such cidofovir and maribavir can be considered Maribavir is approved for treating post-transplant refractory CMV disease in pediatric patients who are at least 12 years old and weigh at least 35 kg (24).

## 3. Epstein-Barr virus

### 3.1. Prevention

**Figure 3:**
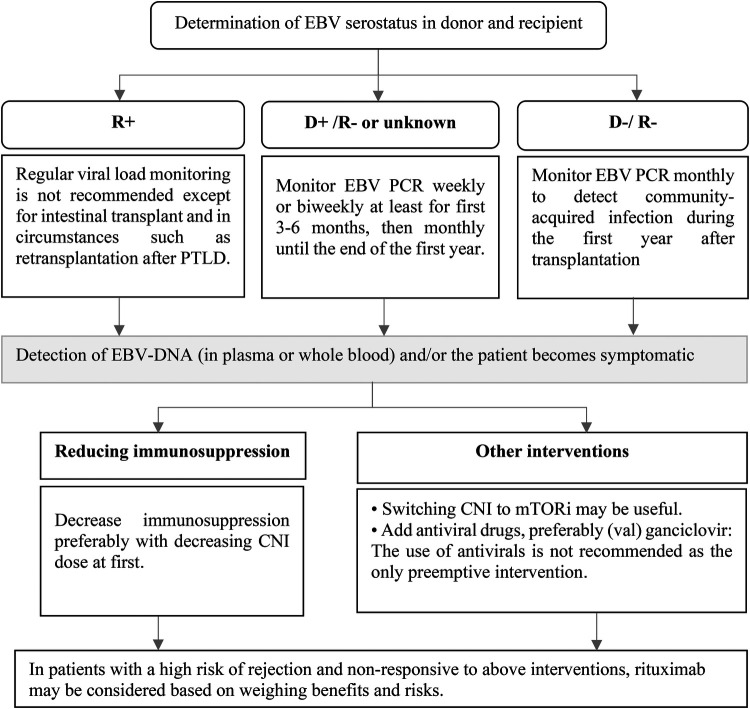
Prevention of EBV infection after pediatric SOT and preemptive intervention. CNI, calcineurin inhibitor; D, donor; DNA, deoxyribonucleic acid; EBV, Epstein–Barr virus; mTORi, mammalian target of rapamycin inhibitors; PCR, polymerase chain reaction; R, recipient.

In high-risk patients (D+/R−), a preemptive strategy is recommended. The standard schedule for monitoring EBV DNA is currently uncertain. The decision on how to monitor viral load should be personalized, taking into account various factors such as the type of organ and the level of ongoing immunosuppression. It is suggested to monitor EBV DNA weekly or every two weeks at least for first 3–6 months, then monthly until the end of the first year. In D−/R− patients, monthly EBV DNA monitoring should be considered due to the risk of community-acquired infection. Patients with changing immunosuppression, rejection events, or those without a viral “set point” may be candidates for ongoing surveillance after the first year after the transplantation. Regular viral load monitoring is not recommended for EBV seropositive SOT recipients, except for intestinal transplant patients and those who are undergoing retransplant following post-transplant lymphoproliferative disorder (PTLD) (Figure 3) (7). Administration of antivirals as a preventive strategy for EBV infection remains controversial. Some retrospective studies of SOT have shown that antiviral drugs are not effective in reducing the incidence of EBV infection and/or PTLD (25, 26). On the other hand, a number of studies have supported the use of antiviral medications after transplantation to reduce the risk of EBV infection (27–29) or EBV disease including PTLD (30). According to a meta-analysis, the incidence of PTLD was unaffected by the prophylactic and preemptive use of antivirals in EBV seronegative patients At the time of transplantation (31). The benefits of IVIG or adoptive immunotherapy using donor-derived cloned EBV-specific cytotoxic T cells for the prevention of PTLD are not clear, so the American Society of Transplantation (AST) does not recommend their use as universal prophylaxis measures (7).

### 3.2. Treatment

If EBV-DNA is detected in plasma or whole blood during routine monitoring or whenever the patient becomes symptomatic, preemptive measures should be considered. Reduction in immunosuppression is the preferred intervention (7, 32). Although there is no specific protocol to reduce the patient's immunosuppression, it is suggested that the dose of calcineurin inhibitor (CNI) is reduced initially (33). Conversion from CNI to a mammalian Target Of Rapamycin (mTOR) inhibitor may also be effective due to their *in vitro* antiproliferative and antiviral properties, although clinical studies supporting their use are lacking (Figure 3) (7, 34). The use of antiviral agents as the only preemptive intervention is not recommended and it can be considered along with other treatments (7). Rituximab administration as preemptive therapy to prevent the consequence of EBV disease such as PTLD cannot be routinely recommended (32). Due to insufficient data, AST guideline does not make a recommendation for or against the use of rituximab in patients not responding to the reduction in immunosuppression (7). Most studies on the use of rituximab as a preemptive intervention have been conducted in hematopoietic stem cell transplant (HSCT) recipients (35, 36) and data on the usefulness of rituximab in SOT patients are limited. In a small retrospective study on 6 pediatric heart recipients who developed primary EBV DNAemia, administration of rituximab resulted in an 83.3% response rate (37). In another prospective single-center study on 8 heart transplantation recipients, a single dose of rituximab reduced PTLD incidence in patients with EBV infection who had a very high viral load or did not respond to reduction of immunosuppression compared with historical controls (38). When weighing the risks and benefits, rituximab administration may be considered in patients who are at high risk for rejection and are not responsive to the reduction of immunosuppression.

## 4. Herpes simplex virus

### 4.1. Prevention

**Figure 4:**
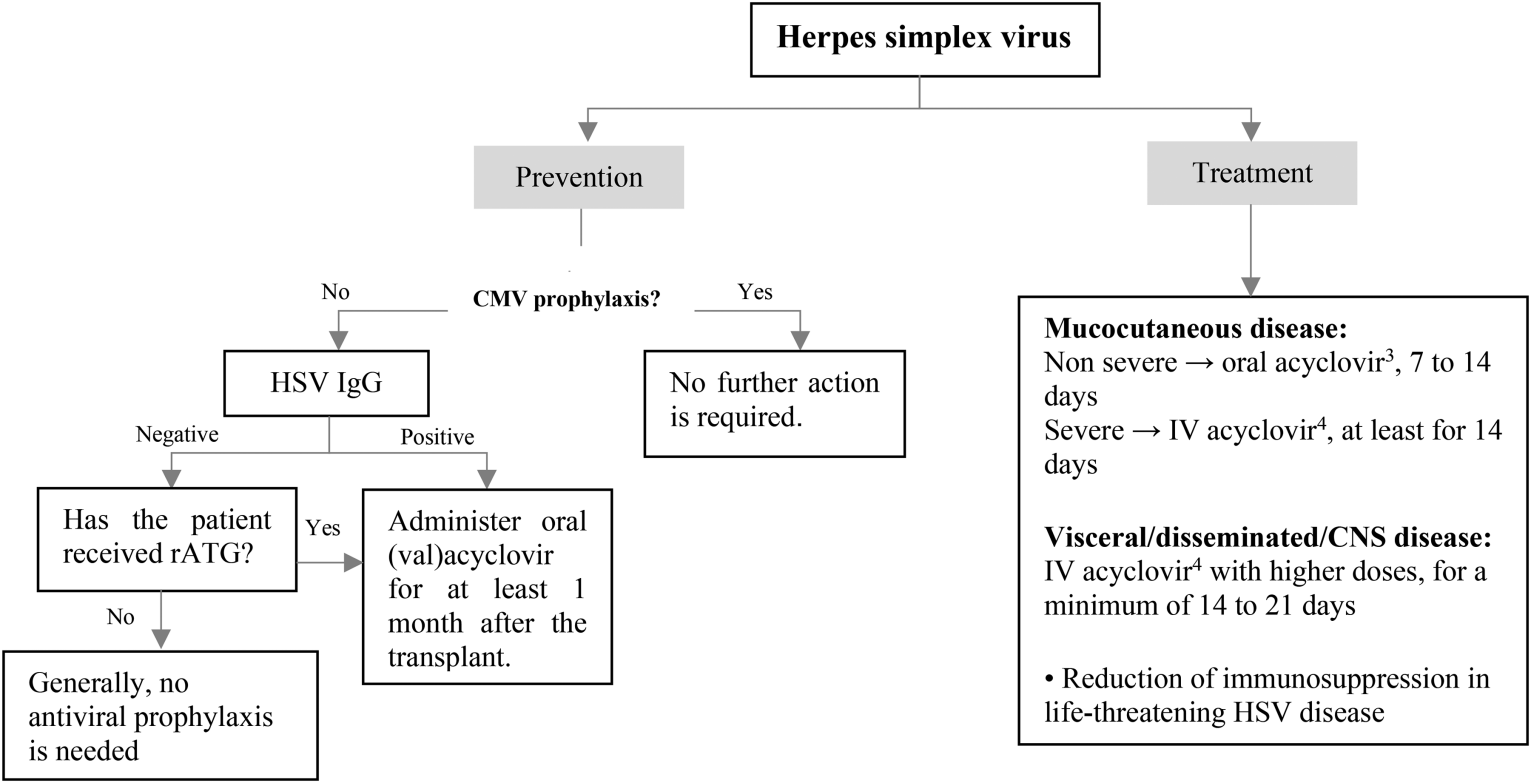
Prevention and treatment of HSV infection after pediatric SOT. CMV, cytomegalovirus; CNS, central nervous system; HSV, herpes simplex virus; intravenous; IgG, immunoglobulin G; rATG, rabbit antithymocyte globulin. ^1^Acyclovir: 200 mg/dose, 3–5 times/day in normal kidney function. ^2^Valacyclovir: <40 kg: 250 mg every 12 h, ≥40 kg: 500 mg every 12–24 h in normal kidney function. ^3^20 mg/kg/dose every 6 h in normal kidney function. ^4^10–15 mg/kg/dose every 8 h in normal kidney function.

Patients taking antiviral medications to prevent CMV infection do not need additional intervention for HSV prophylaxis. Pediatric SOT recipients who are seropositive for HSV-1 and HSV-2 and are not administered antiviral agent for CMV prophylaxis should receive HSV-specific prophylaxis for a minimum of one month. In cases where the recipient is HSV seronegative but the donor is HSV seropositive, which is not uncommon in pediatric patients, some practitioners may consider administering antiviral prophylaxis. In addition, for patients receiving anti-rejection therapy with T-cell depleting drugs, an antiviral agent with activity against HSV can be resumed in the absence of anti-CMV prophylaxis (Figure 4) (39).

### 4.2. Treatment

HSV disease in immunocompromised children may be treated with oral or intravenous acyclovir. While both acyclovir and valacyclovir are considered equally effective when administered at the appropriate doses, there is still limited clinical experience with valacyclovir in pediatric patients. For non-severe mucocutaneous disease, oral acyclovir can be given for a period of 7–14 days. Patients with more serious mucocutaneous illness or those with disseminated/ central nervous system (CNS) disease should receive intravenous acyclovir with higher doses for a minimum of 14–21 days. In cases of life-threatening HSV disease, reducing immunosuppression should be considered (Figure 4).

## 5. Varicella-Zoster virus

### 5.1. Vaccination

**Figure 5:**
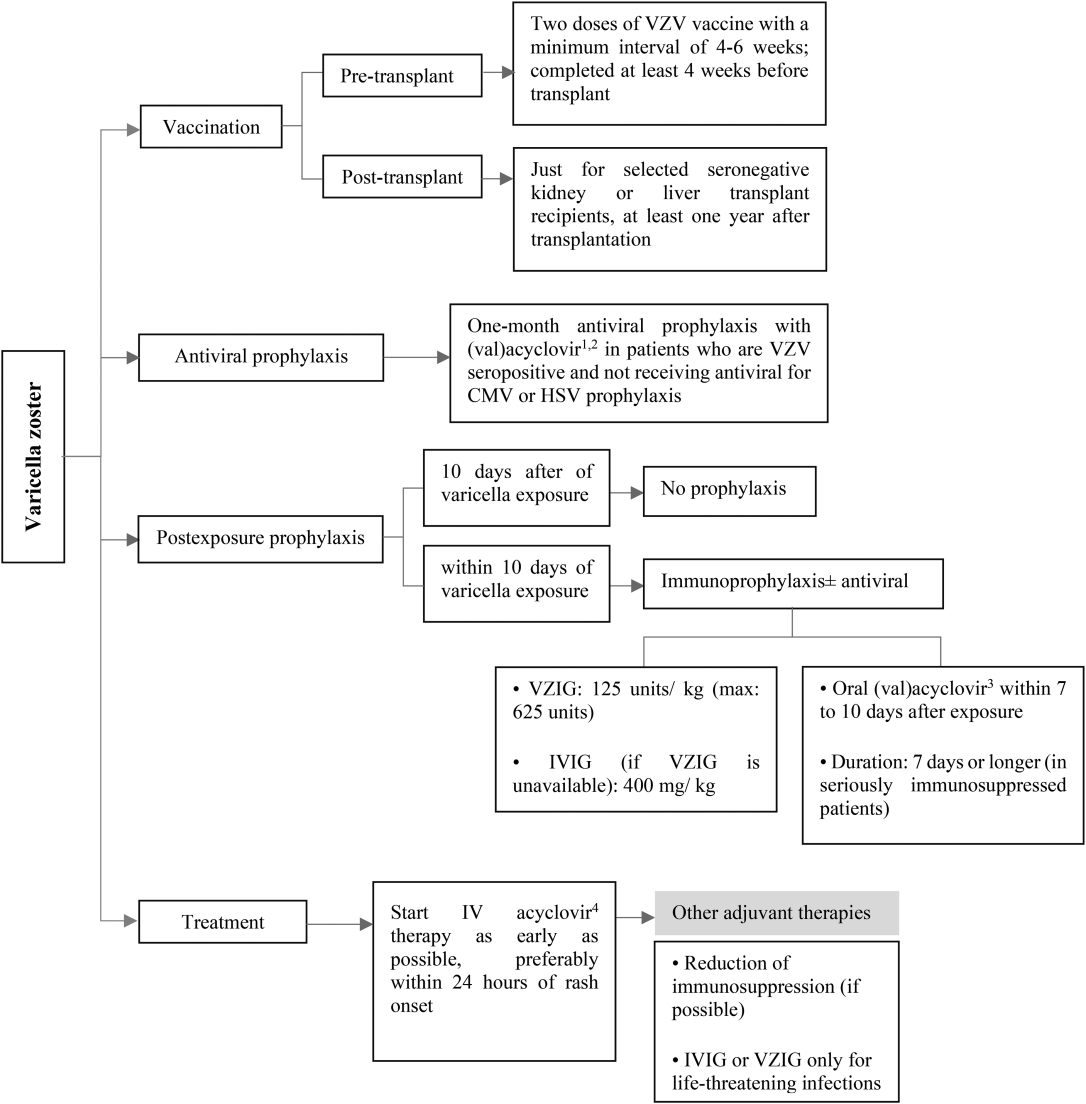
Prevention and treatment of VZV infection after pediatric SOT. CMV, cytomegalovirus; HSV, herpes simplex virus; intravenous; IV, intravenous; IVIG, intravenous immunoglobulin; VZIG, varicella zoster immune globulin; VZV, varicella zoster virus. ^1^Acyclovir: 200 mg/dose, 3–5 times/day in normal kidney function. ^2^Valacyclovir: <40 kg: 250 mg every 12 h, ≥40 kg: 500 mg every 12–24 h in normal kidney function. ^3^Acyclovir: 20 mg/kg/dose every 6 h and valacyclovir: 20 mg/kg/dose every 8 h in normal kidney function. ^4^10 mg/kg/dose every 8 h in normal kidney function.

Varicella vaccination should be considered in seronegative SOT candidates who do not have contraindications to receive live attenuated vaccine (40, 41). In order to improve response rates to varicella vaccination, two doses of the vaccine should be considered with a minimum interval of 4–6 weeks (40, 42, 43). Varicella vaccine should be given at least 4 weeks before the transplantation surgery (Figure 5). Live vaccines are contraindicated after the transplantation, but some selected seronegative kidney and liver transplant recipients with stable conditions may be eligible to receive the varicella vaccine, at least one year after transplantation [see ref (44) for more information]. A significant decrease in VZV antibody levels has been observed following transplantation, particularly among individuals who received fewer pre-transplant vaccine doses (45, 46). However, routine assessment of VZV serology to guide booster dosing is not recommended, as commercial assays have lower sensitivity in detecting vaccine-induced immunity (47).

### 5.2. Prevention

#### 5.2.1. Antiviral prophylaxis

For those SOT recipients who are seropositive for VZV and not taking antiviral medication for CMV or HSV prophylaxis (which is a rare situation), it is recommended to consider administering (val)acyclovir for at least one month after the transplantation (Figure 5) (9, 40, 48).

#### 5.2.2. Postexposure prophylaxis

Post-exposure prophylaxis should be considered in seronegative pediatric SOT recipients after a significant exposure. It is recommended to use immunoprophylaxis with Varicella-zoster immune globulin (VZIG) or non-specific IVIG (as an alternative if VZIG is not available) as early as possible, with a maximum lag of 10 days after exposure (40, 49–52). Antivirals can be considered as an adjunct in patients who are receiving immunoprophylaxis (40, 49). In addition, antiviral drugs should be administered to the patients who are unable to receive VZIG (40, 53). Preemptive therapy with (val)acyclovir should be started within 7–10 days after VZV exposure for a treatment duration of 7 days (40, 52). Extended duration to 28 may be considered in severely immunosuppressed patients (Figure 5) (40).

### 5.3. Treatment

Post-transplant patients have a higher risk of developing severe and disseminated VZV disease, so early initiation of intravenous acyclovir therapy, particularly within 24 h of rash onset, is recommended to maximize treatment benefit (40, 52). The American Society of Transplantation recommends against the routine use of immune globulin preparations, including IVIG or VZIG, for the treatment of VZV infection. These treatments may only be considered anecdotally for life-threatening infections (40). Reduction of patient's immunosuppression may be considered (Figure 5). In children with varicella who are taking aspirin to prevent early thrombotic events after transplantation or other indications, aspirin should be stopped due to the risk of Reye syndrome (52).

## 6. Influenza

Influenza is the most common vaccine-preventable infection affecting SOT recipients and results in considerable healthcare utilization (5). Influenza is linked with significant hospitalization rates, lower respiratory tract infections, mechanical ventilation requirements, and even mortality in SOT recipients. Annual influenza vaccination and prompt initiation of antiviral therapy improves the clinical outcomes of Influenza infected SOT patients (54).

### 6.1. Prevention

#### 6.1.1. Vaccination

**Figure 6:**
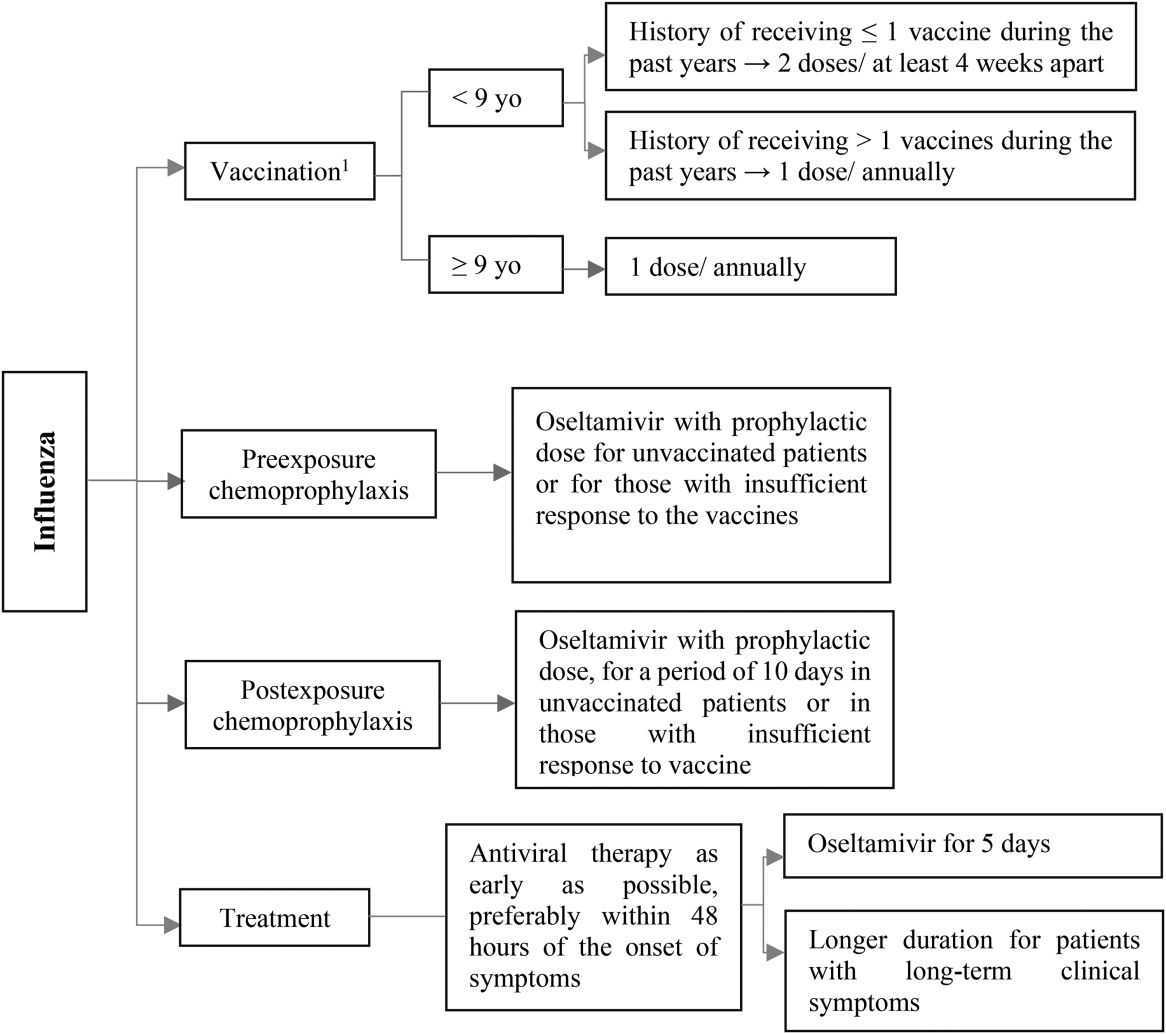
Prevention and treatment of influenza infection after pediatric SOT. yo, years old. ^1^Children who are 36 months of age or older should receive a 0.5-ml dose of IIV, while children aged between 6 and 35 months may receive either a 0.25-ml or 0.5-ml dose based on the manufacturer's recommendations.

Annual influenza vaccination is highly recommended for SOT patients age 6 months and older, as it serves as the primary method of preventing influenza and its associated complications. Close contacts of SOT recipients, especially those residing in the same household, should undergo yearly influenza immunization (55). Three types of influenza vaccines are available, including inactivated influenza vaccine [IIV], recombinant influenza vaccine [RIV], and live attenuated influenza vaccine (LAIV) (56). LAIV (intranasal vaccine) is not recommended post-transplantation due to the possible risk of developing vaccine-related viral disease (57). RIV is still not approved in pediatric population (58). The SOT pediatrics can receive any licensed, age-appropriate IIV at least one month after the transplantation (Figure 6). Alternative methods and formulations including high-dose or adjuvanted vaccines, and booster shots can enhance immunity without any major safety concerns in SOT populations (59), but limited studies have investigated these strategies specifically in pediatrics (57).

#### 6.1.2. Preexposure chemoprophylaxis

Preexposure antiviral prophylaxis with oseltamivir may be considered for children age 3 months or older who have not received the influenza vaccine due to contraindications, unavailability, or expected low effectiveness (e.g., high immunosuppression due to acute rejection treatment or early months after transplantation) during the flu season or for a period of 12 weeks (60–62).

#### 6.1.3. Postexposure chemoprophylaxis

Postexposure antiviral prophylaxis with oseltamivir is suggested for SOT recipients aged more than 3 months who have close contacts with a patient with confirmed influenza (especially in situations of nosocomial influenza and in severely immunosuppressed patients). A period of 10 days has been suggested for postexposure chemoprophylaxis (55, 61).

### 6.2. Treatment

All SOT children with confirmed or suspected influenza should take antiviral treatment as soon as possible after symptom onset, preferably within 48 h (61). Oral oseltamivir is the preferred medication for treatment of influenza due to more information on its effectiveness in pediatric transplant recipients (9). The usual duration of antiviral therapy with oseltamivir is 5 days; an extended duration may be needed in patients with long-term clinical symptoms (Figure 6) (61, 62). Alternative antiviral options include inhaled zanamivir for children 7 years or older, intravenous peramivir for children 2 years or older, and oral baloxavir for children 12 years or older weighing at least 40 kg (52). Experiences with zanamivir, peramivir, and baloxavir are lacking in pediatric transplant patients. Intravenous formulations (zanamivir or peramivir) may be used in severely ill patients or those unable to tolerate oral drugs (9). In addition, antiviral therapy should be considered for symptomatic individuals who reside with SOT recipients, particularly severely immunocompromised ones (52, 62).

## 7. Hepatitis B virus (HBV)

Hepatitis B virus (HBV) infection significantly increases the risk of hepatic dysfunction in SOT recipients (63). Liver transplantation in the context of HBV-induced cirrhosis or hepatocellular carcinoma is rare in children (64). The importance of HBV in pediatric SOT is due to the risks associated with HBV reactivation or *de novo* infection (65). The risk of HBV reactivation or *de novo* infection in a SOT recipient who does not receive antiviral treatment or prophylaxis is determined by the HBV status of both the donor and the recipient (66). The routine screening for HBV using HBsAg, anti-HBs, and anti-HBc testing was previously recommended for all children at risk of HBV reactivation. However, according to the latest U.S. Public Health Service Guideline, pre-transplant HBV testing is no longer necessary for pediatric SOT candidates under the age of 12 who have already undergone infectious disease testing after birth (67).

### 7.1. Prevention of *de novo* or recurrent HBV infection post- transplantation

The risk of HBV transmission through organ donation is higher in liver transplantation, and it is significantly lower in non-liver transplantation (68). Most pediatric liver transplant candidates have not been exposed to HBV at the time of transplantation, so they are at a greater risk for developing *de novo* infections if they receive organs from anti-HBc-positive donors (69). The use of antiviral agents reduces the rate of *de novo* HBV infection.

**Figure 7:**
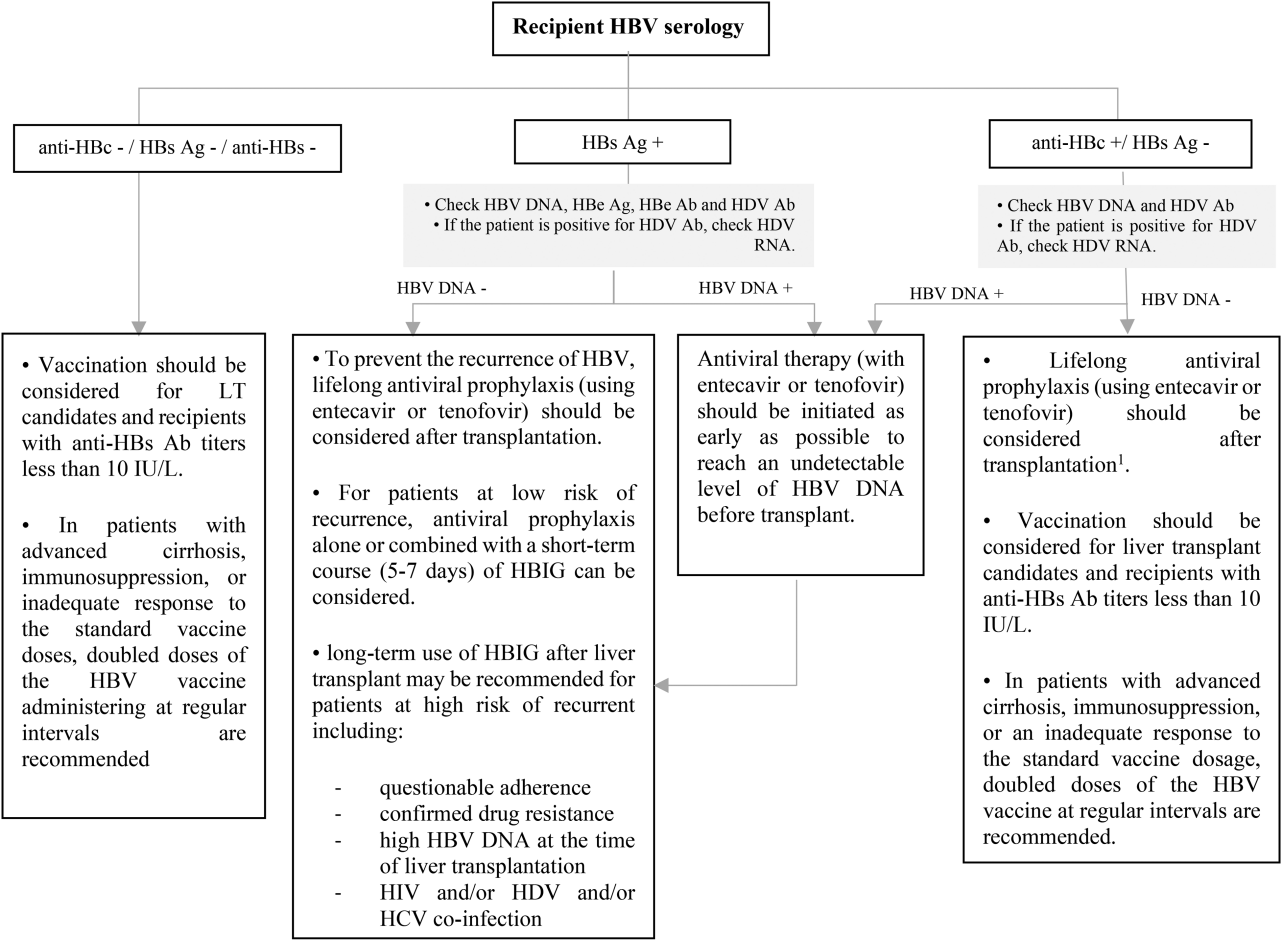
Approach to management of HBV in liver transplant candidates or recipients according to recipient HBV serology. Ab, antibody; Ag, antigen; DNA, deoxyribonucleic acid; HBe, hepatitis B e; HBc, hepatitis B core; HBs, hepatitis B surface; HBV, hepatitis B virus; HDV, hepatitis D virus; HBIG, hepatitis B immune globulin; HCV, hepatitis C virus; HIV, human immunodeficiency virus; LT, liver transplantation; RNA, ribonucleic acid. ^1^This recommendation is based on ESPGHAN guideline, while the AST guideline does not recommend for the using of antiviral prophylaxis in this context unless for patients with intense immunosuppression such as patients who received lymphocyte depleting agents.

**Figure 8:**
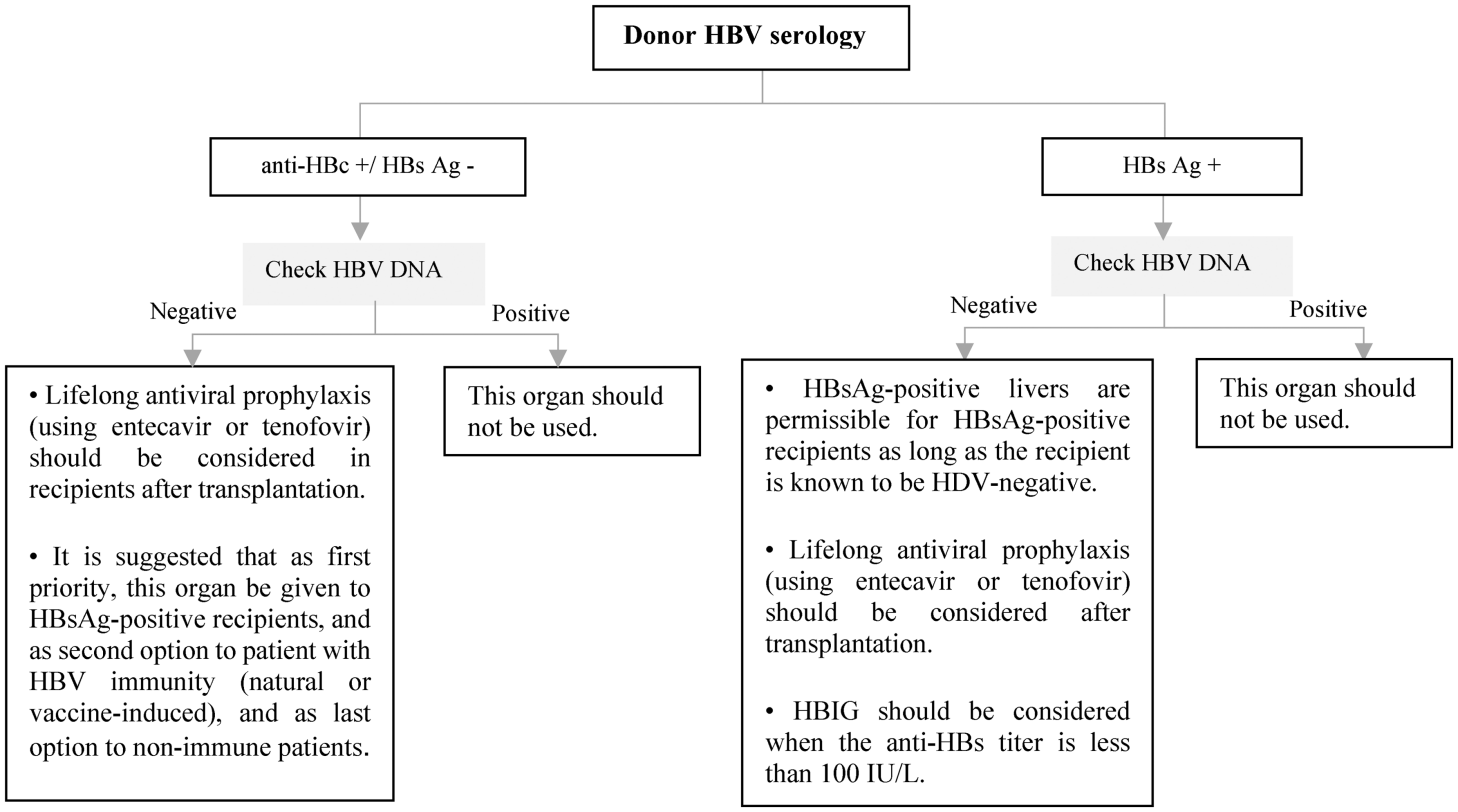
Approach to management of HBV in liver transplant candidates or recipients according to doner HBV serology. Ag, antigen; DNA, deoxyribonucleic acid; HBc, hepatitis B core; HBs, hepatitis B surface; HBV, hepatitis B virus; HDV, hepatitis D virus; HBIG, hepatitis B immune globulin.

**Figure 9:**
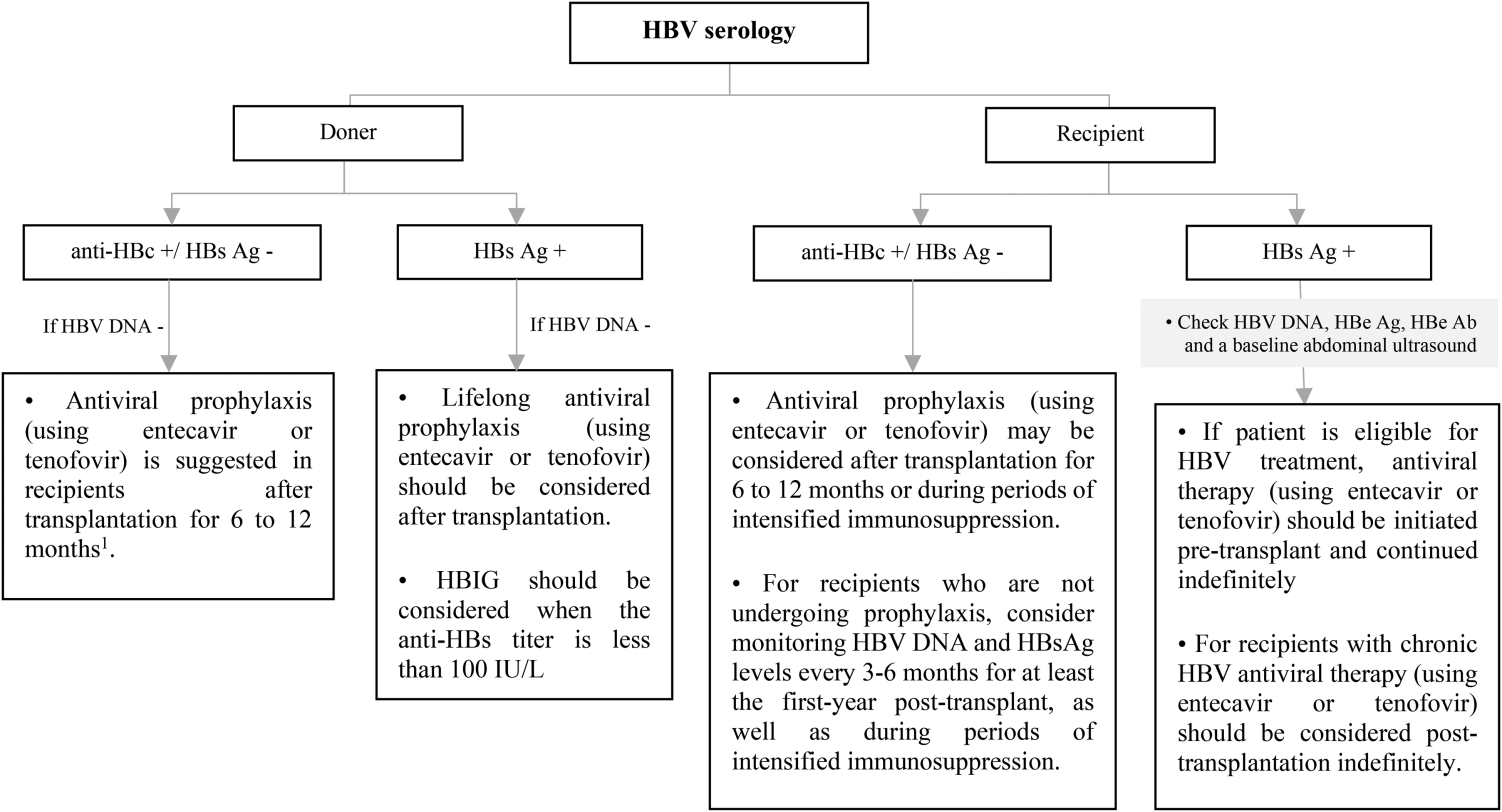
Approach to management of HBV in non-liver SOT transplant candidates or recipients according to recipient and doner HBV serology. Ab, antibody; Ag, antigen; DNA, deoxyribonucleic acid; HBe, hepatitis B e; HBc, hepatitis B core; HBs, hepatitis B surface; HBV, hepatitis B virus; HDV, hepatitis D virus; HBIG, hepatitis B immune globulin. *Vaccination should be considered for HBV-uninfected, non-immune SOT candidates or recipients. ^1^This recommendation is based on ESPGHAN guideline, while the AST guideline recommends antiviral prophylaxis is not necessary for in HBV immune recipients and it should be considered only in non-immune (anti-HBs -) recipients up to 1-year after transplantation.

All pediatric recipients with a history of HBV infection receive prophylaxis or continue antiviral therapy if they have positive HBV DNA at the time of transplantation to prevent HBV recurrence. Algorithmic approaches to management of HBV in livre and non-liver transplant are shown in Figures 7–9 (63, 70, 71).

### 7.2. Antiviral selection

The Hepatology Committee of the European Society for Pediatric Gastroenterology, Hepatology and Nutrition (ESPGHAN) guideline suggests that antiviral drugs with a high barrier to resistance, such as entecavir or tenofovir, should be used instead of lamivudine for prophylaxis, pre-emptive treatment, and the treatment of HBV reactivation in pediatric patients (71). Two formulations of tenofovir are available, tenofovir disoproxil fumarate (TDF) and tenofovir alafenamide (TAF). It seems that renal and bone toxicity is lower with TAF (72, 73). TAF is approved for use in patients 12 years of age or older with chronic HBV, while the use of TDF and entecavir are approved in children 2 years of age or older weighing at least 10 kg.

## 8. Severe acute respiratory syndrome-coronavirus-2 (SARS-COV-2) infection

### 8.1. Vaccination

Unless there are any contraindications, all transplant recipients are eligible for coronavirus disease 2019 (COVID-19) vaccination. Administering vaccines has been shown to offer a degree of safeguarding against COVID-19 infections and could potentially also serve as a protective measure towards the emergence of post-COVID sequelae in children, such as immune-mediated multisystem inflammatory syndrome (MIS-C) and post-COVID syndrome, which is also known as “long COVID” (74). In comparison to the general population, adult SOT recipients exhibit reduced immunogenicity and efficacy of COVID-19 vaccines (75). Conversely, results from pediatric studies reveal a somewhat moderate increase in COVID-19 vaccine immunogenicity when compared to adult SOT recipients, but the observed level remains lower than that seen in healthy children (76–79).

Although monovalent vaccines have been used since the introduction of mRNA COVID-19 vaccines for patients, after April 2023 only bivalent vaccines including Pfizer-BioNTech and Moderna COVID-19 Vaccine are authorized for persons ≥6 months of age in the United States. Centers for Disease Control and Prevention (CDC) suggests administering three bivalent mRNA doses to unvaccinated individuals aged ≥6 months who have moderate or severe immunodeficiency, including SOT recipients. Individuals aged 6 months or older who previously received only monovalent doses are advised to receive either one or two bivalent mRNA vaccine doses based on their age and the vaccine product used. Those who have already received bivalent mRNA vaccine doses can opt to get one or more additional doses (80). To access more details about COVID-19 vaccination schedule in immunocompromised patients please refer to CDC guidance (80).

The optimal schedule for administering vaccines in the post-transplantation context remains uncertain. To ensure effectiveness, COVID-19 vaccines should be given at least 14 days before transplantation. If pre-transplantation vaccination is unfeasible, it is recommended to delay it by at least one month after the transplantation procedure. In individuals treated with T-cell depleting agents (such as anti-thymocyte globulin) or B-cell depleting agents (such as rituximab), vaccine administration should be postponed for a minimum of three months (81–83). Patients who are on continuous B-cell-depleting therapies should receive COVID-19 vaccines around four weeks before their next scheduled therapy (80).

### 8.2. Treatment

**Figure 10:**
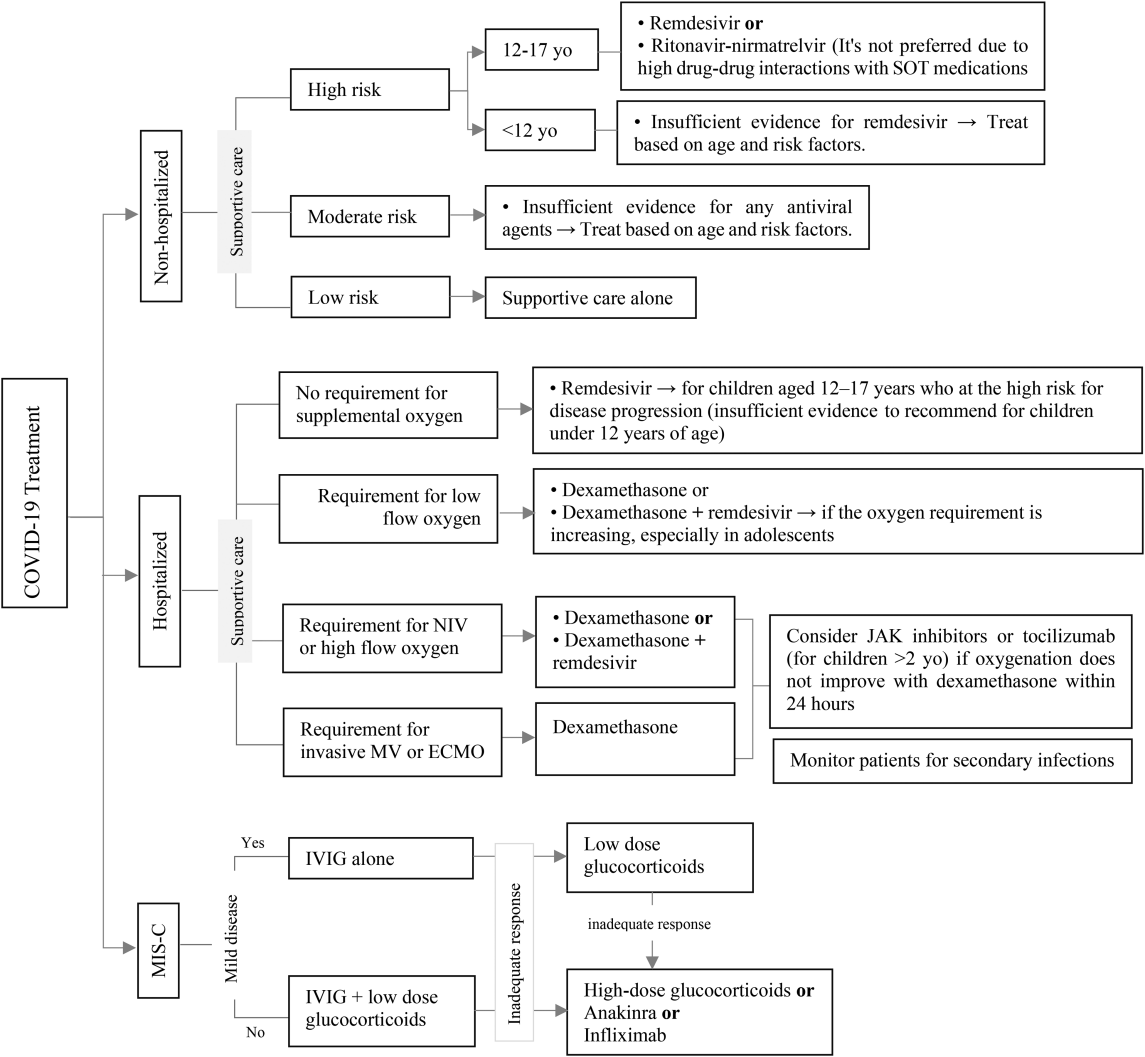
Approach to management of COVI-19 infection in pediatric SOT recipients. ECMO, extracorporeal membrane oxygenation; JAK, Janus kinase; IVIG, intravenous immunoglobulin; MIS-C, multisystem inflammatory syndrome in children; MV, mechanical ventilation; NIV, non-invasive ventilation; SOT, solid organ transplantation.

Current research provides insufficient evidence regarding the optimal management of COVID-19 in pediatric transplant recipients. The optimal therapeutic approach for COVID-19 in this particular population remains unknown. Consequently, the standard procedures for evaluating and managing COVID-19 in nontransplant patients may be applicable for transplant recipients (81). Figure 10 shows an overview of the treatment of COVID-19 in the pediatric population based on the Infectious Diseases Society of America (IDSA) and National Institutes of Health (NIH) guidelines (81, 84). It's important to note that certain antiviral drugs, specifically Nirmatrelvir-ritonavir and Janus kinase (JAK) inhibitors, exhibit substantial drug-drug interactions with immunosuppressive drugs which are frequently administered to SOT recipients. If these immunosuppressive agents persist during COVID-19 infection, it is essential to conduct frequent monitoring of their blood levels and consider adjusting the dose to maintain optimal immunosuppression (85, 86).

### 8.3. Immunosuppression modification

According to the data collected from pediatric patients who underwent SOT, immunosuppression did not pose a supplementary risk for severe or complicated COVID-19 cases. The majority of patients demonstrated mild to moderate levels of disease severity, which is similar to that observed in the general population (87). A systematic review and meta-analysis suggests that continuing immune suppressive therapy may be safe for SOT recipients with moderate or severe COVID-19 (88), as it can potentially alleviate the cytokine storm (89). The decision to adjust the patient's immunosuppressive regimen should be approached individually, with careful consideration given to factors such as disease severity, the specific immunosuppressants administered, the type of transplant, the time since transplantation, the concentration of the drug, and the likelihood of graft rejection and superinfection (81). Some experts and transplant centers suggest to decrease or withhold the use of antimetabolites in patients with lymphopenia who have developed moderate to severe COVID-19, while continuing to administer CNIs (90).

## 9. Adenovirus

### 9.1. Prevention

Using antiviral drugs as a preventive measure for adenovirus infection is not recommended due to the lack of evidence regarding their effectiveness. Therefore, prophylaxis is not advised at this time. Routine screening and preemptive strategies are not recommended for adenovirus infection (91).

### 9.2. Treatment

Patients who do not exhibit any symptoms are not advised to receive treatment, whereas those with adenovirus disease (i.e., symptomatic patients) are eligible for treatment. The therapeutic approach involves providing supportive care, reducing immunosuppression, and administering antiviral therapy (91, 92). Supportive care may include the replacement of fluids and electrolytes in dehydrated patients, as well as the use of appropriate medications to manage diarrhea, nausea, and vomiting (92). There is currently no agreement on which immunosuppressive drug should be stopped or reduced, or when it should be resumed (91). Antiviral medications can be initiated either at the same time as adjusting immunosuppression or afterwards. Cidofovir is the most prescribed antiviral in transplant centers as the standard treatment for severe, progressive, or disseminated adenovirus disease in SOT recipients (91). There are no clinical trials supporting the use of cidofovir in pediatric patients, and there are only a few retrospective studies with controversial results (93–96). In a more recent study about the use of cidofovir for the treatment of adenovirus infection in hematopoietic and solid organ pediatric transplant recipients, the benefits of cidofovir could not be established (96). Cidofovir can be administered in two ways: 1 mg/kg three times per week for 2 weeks or 5 mg/kg per week for 2 weeks, followed by maintenance therapy with 5 mg/kg every other week (91, 97). Maintenance therapy should continue until all symptoms have disappeared, and three consecutive negative samples, taken one week apart from the initially positive sites, have been documented (91). The most significant adverse effects of cidofovir are nephrotoxicity and neutropenia. To minimize nephrotoxicity, it is recommended to administer cidofovir alongside oral probenecid and intravenous normal saline hydration (91). Brincidofovir is a lipid-conjugated prodrug of cidofovir that has been shown to have a lower risk of nephrotoxicity compared to cidofovir and to have more potent *in vitro* activity against adenoviruses, but it is not currently available commercially (92).

## 10. BK polyomavirus

BK polyomavirus (BKPyV) is primarily responsible for polyomavirus-associated nephropathy (PyVAN) following kidney transplantation. However, BKPyV-related complications are infrequent in other types of solid organ transplantations (98). Children face a higher risk of BKPyV-related complications due to a higher rate of BKPyV-seronegativity at the time of transplantation (99, 100).

### 10.1. Screening and diagnosis in kidney recipients

**Figure 11:**
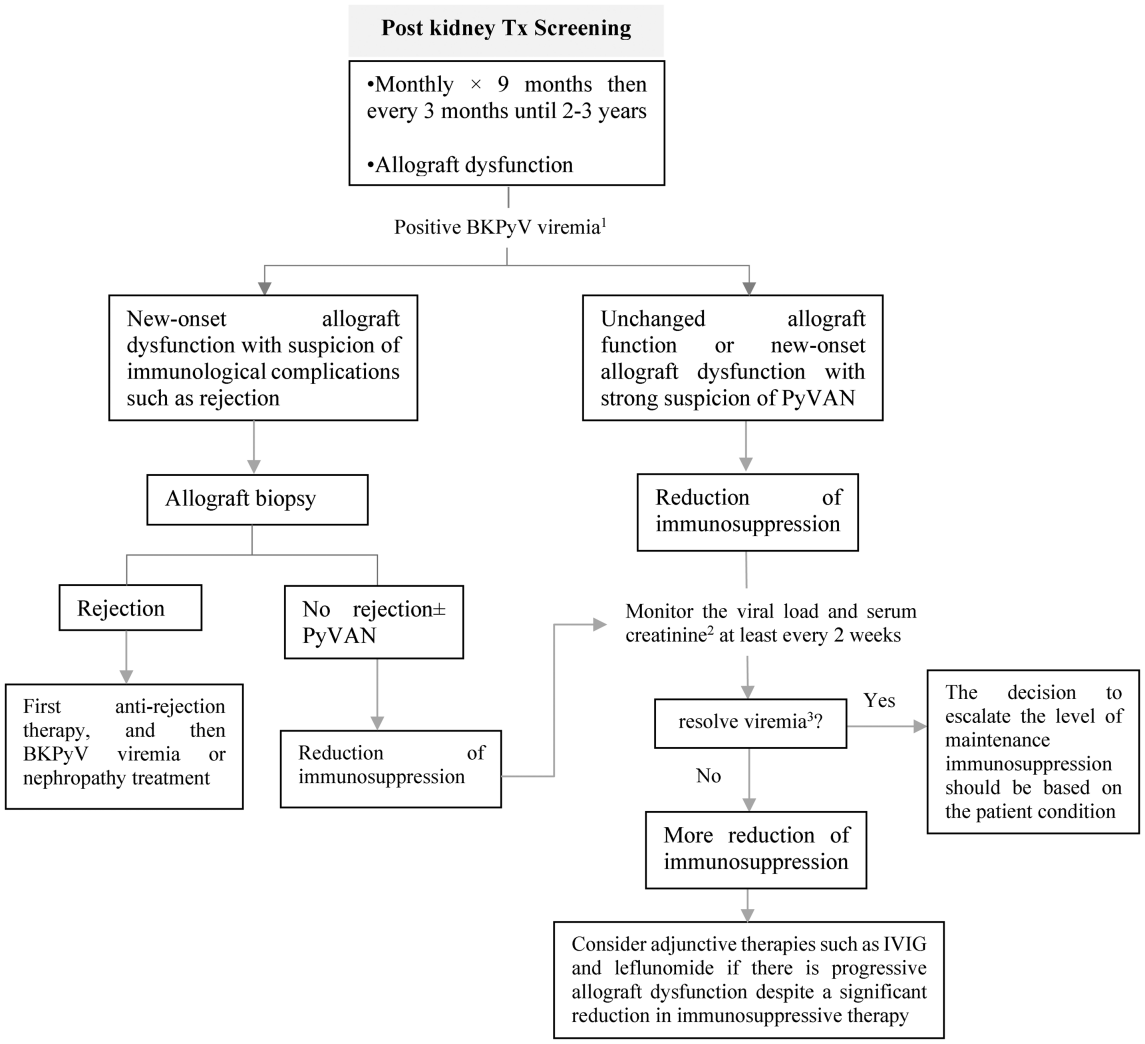
Approach to screening and treating BKPyV in pediatric kidney transplant recipients. PyVAN, polyomavirus-associated nephropathy; IVIG, intravenous immunoglobulin. ^1^To confirm BKPyV viremia, it is essential to have either sustained viral load above 1,000 copies/ml in two consecutive measurements within a 3-week period or an increase to over 10,000 copies/ml in one of two measurements. ^2^If the serum creatinine level rises by 25% or more from its initial level during immunosuppression reduction, acute rejection should be evaluated. ^3^Two consecutive undetectable plasma viral load tests, performed at least one week apart.

The preferred screening test for BKPyV is quantitative plasma PCR due to its high sensitivity, specificity, and positive predictive value (98, 101). The use of urine BKPyV PCR as a screening test is not generally recommended due to concerns about specificity and cost. If a positive result is obtained from the urine test, confirmation with plasma PCR is necessary (102). Furthermore, monitoring the response to therapy through urine BKPyV PCR is less effective compared to plasma because the decrease in immunosuppression shows a delay in changes in urine viral load compared to plasma viral load (103). AST guideline recommended to screen on a monthly basis for the first 9 months, and then every 3 months for the following 2 years (Figure 11) (98). In pediatric recipients, extending screening every three months until the third-year post-transplant may be useful (100).

Biopsy is considered the gold standard for the diagnosis of BKPyV-associated nephropathy as it enables the detection of any underlying conditions or complications. According to the AST guideline, biopsy should primarily be considered for patients with BKPyV viremia who are experiencing new-onset kidney dysfunction or who have markers indicating an elevated risk of immunological complications (98).

### 10.2. Treatment

Treatment should be considered for patients with BKPyV-associated nephropathy or those with sustained BKPyV viremia. The primary approach is to reduce immunosuppression therapy (98, 101). This can be achieved by gradually reducing or discontinuing antiproliferative or calcineurin inhibitors. However, there is currently no standardized approach to reducing immunosuppression, as transplant centers typically have their own unique protocols (104). In cases where patients continue to experience progressive allograft dysfunction despite a significant reduction in immunosuppressive therapy over several weeks to months, the addition of IVIG may be considered (105, 106). In such situations, other adjunctive therapies such as leflunomide, cidofovir, and quinolone antibiotics could also be explored (Figure 11) (98).

## 11. Conclusion

In conclusion, viral infections remain a significant factor affecting the outcomes of pediatric organ transplantation. Some viral infections, such as CMV and EBV, are more important in children than adults due to their higher rate of seronegativity. Prevention and treatment of viral infections can reduce morbidity and mortality associated with these infections and improve the long-term outcomes for pediatric organ recipients. preventative measures including vaccination, antiviral prophylaxis and preemptive therapy have been shown to be effective in reducing the incidence of viral diseases. Close monitoring and prompt treatment of viral infections are crucial for successful outcomes. To further improve prevention and treatment strategies for viral infections in this population, ongoing research efforts are necessary. By identifying the most effective approaches to preventing and treating viral infections in pediatric organ recipients, we can continue to improve their overall health and well-being.
